# Formation and Luminescent Properties of Al_2_O_3_:SiOC Nanocomposites on the Base of Alumina Nanoparticles Modified by Phenyltrimethoxysilane

**DOI:** 10.1186/s11671-017-2245-z

**Published:** 2017-08-01

**Authors:** D. V. Kysil, A. V. Vasin, S. V. Sevostianov, V. Ya Degoda, V. V. Strelchuk, V. M. Naseka, Yu. P. Piryatinski, V. A. Tertykh, A. N. Nazarov, V. S. Lysenko

**Affiliations:** 1 Lashkaryov Institute of Semiconductor Physics of the NAS of Ukraine, Kyiv, 03680 Ukraine; 2 Institute of Surface Chemistry of the NAS of Ukraine, Kyiv, Ukraine; 3 Taras Shevchenko National University, Kyiv, Ukraine; 4 Institute of Physics, NAS of Ukraine, Kyiv, Ukraine; 5 National Technical University of Ukraine “Igor Sikorsky Kyiv Polytechnic Institute”, Kyiv, Ukraine

**Keywords:** Fumed alumina, Al_2_O_3_:SiOC nanocomposite, Phenyltrimethoxysilane, Photoluminescence

## Abstract

Al_2_O_3_:SiOC nanocomposites were synthesized by thermal treatment of fumed alumina nanoparticles modified by phenyltrimethoxysilane. The effect of annealing temperature in inert ambient on structure and photoluminescence of modified alumina powder was studied by IR spectroscopy as well as photoluminescence spectroscopy with ultraviolet and X-ray excitation. It is demonstrated that increase of annealing temperature results in formation of silica precipitates on the surface of alumina particles that is accompanied by development and spectral evolution of visible photoluminescence. These observations are discussed in terms of structural transformation of the surface of Al_2_O_3_ particles.

## Background

Recently, it was reported that silica nanoparticles with surface carbonized by pyrolysis of phenylmethoxy groups exhibit strong visible photoluminescence (PL) under ultraviolet excitation [1]. Materials that demonstrate effective broad band visible PL at room temperature without heavy metal activators are of great interest as a potential alternative to expansive rare earth-doped ceramic phosphors for artificial white light sources on the base of compact gas-discharge lamps and light-emitting diodes. Though similar SiO_2_:C materials have been reported previously to demonstrate broadband visible PL [2–5], origin of light emission centers is unclear until now. One of the basic hypothesis associates emission centers in SiO_2_:C with carbon nanoclusters [1, 2, 5]. In frame of this model, SiO_2_ nanopowder can be considered as morphological template with high specific surface area that provides high concentration of carbon-related emission centers located on the silica surface. Verification of this hypothesis obviously needs further study of luminescent properties of carbonized surface in related nanostructured materials. Fumed alumina is a well candidate as morphological template with relatively high specific surface area. Excellent mechanical properties, good chemical inertness, and electronic structures make alumina-based ceramics widely used as high-temperature functional materials in electrical and optical devices [6–11]. Optically and X-ray excited PL in superfine Al_2_O_3_ powder with intentionally carbonized surface of nanoparticles is analyzed in the present report. Procedure of surface carbonization was similar to that used for carbonization of fumed silica in [1], i.e., successive procedure of chemical grafting of phenylmethoxy groups to the surface of nanoparticles followed by thermal calcinations in chemically inert ambient.

## Methods

Pyrogenic Al_2_O_3_ powder (89 m^2^/g, particle size 30–50 nm) was treated with a phenyltrimethoxysilane (PhTMS) toluene solution (1.73 ml of PhTMS per 10 ml of toluene) at 70 °C for 4 h in the presence of triethylamine as a catalyst. The aim of this procedure is grafting of phenylmethoxy groups to the alumina surface. The reaction product (hereafter “phenyl-alumina”) was dried and subjected to thermal annealing at temperatures of 400, 500, 600 °С, for 30 min in flow of pure nitrogen at atmospheric pressure.

Interatomic bonding was studied by IR spectroscopy. The Fourier transform infrared (FTIR) analysis was performed in transmission mode using vacuum Bruker Vertex 70 V. The FTIR spectra were recorded at room temperature in spectral range of 400–5000 cm^−1^ using the KBr sample tablets. Photoluminescence was studied under ultra-violet (290 nm) and X-ray (13–14 keV) excitation. Photoluminescence under ultra-violet excitation was studied using excitation by a 290-nm semiconductor laser (5 mW). The spectra were recorded using spectrometer LIFESPEC II (Edinburgh Instruments). The X-ray luminescence was excited by X-ray radiation with energy of 13–14 keV. Radiation of the samples was recorded using a monochromator MDR-2 and a photomultiplier FEP-106.

## Results and Discussion

### IR Spectroscopy

**Fig. 1:**
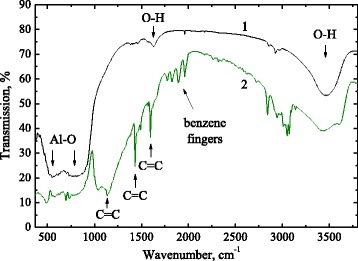
FTIR transmission spectra of pristine fumed alumina before (spectrum 1) and after chemical treatment (spectrum 2). Spectra are offset along the ordinate axis for clarity

FTIR transmission survey spectra of pristine alumina and phenyl-alumina are shown in Fig. 1. Amorphous aluminum oxide structural matrix in pristine alumina is represented by broad absorption bands at 540 and 800 cm^−1^ (Fig. 1, spectrum 1). It is well known that crystalline aluminum oxide exists in a variety of metastable structures (transition aluminas—χ, γ, δ, η, θ) as well as in its stable α-Al_2_O_3_ phase. Structure of metastable polimorphe can be classified in terms of the structure of oxygen anion sublattice (face-centered cubic or a hexagonal close-packed) and the distribution of aluminum cations into this sublattice in tetrahedral (AlO_4_) and/or octahedral (AlO_6_) interstitial sites [12]. In amorphous solid, there is no sense of crystalline polymorphism but vibration properties of local bonds are also determined by Al atom coordination. Two broad bands at 540 and 800 cm^−1^ in FTIR spectrum of pristine fumed alumina (Fig. 1, spectrum 1) can be assigned to mixture of absorption by Al-O stretching vibrations in tetrahedral and octahedral coordination respectively [13, 14].

**Fig. 2:**
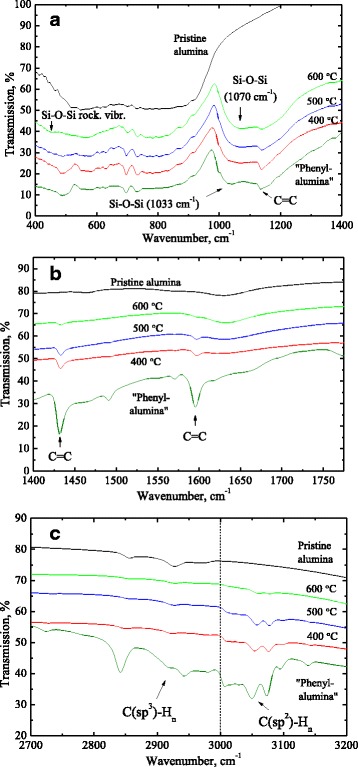
Selected spectral ranges of FTIR spectra of pristine alumina (spectrum 1), and *phenyl-alumina* before and after annealing at 400, 500, and 600 °C in the spectral ranges 400–1400 cm^−1^ (**a**), 1400–1775 cm^−1^ (**b**), and 2700–3200 cm^−1^ (**c**). Spectra are offset along the ordinate axis for clarity

The broad absorption band in the range of 3000–3800 cm^−1^ and the narrow band at 1630 cm^−1^ (Fig. 2) are attributed to stretching and bending vibration modes of O–H bonds respectively due to both surface hydroxyl groups in Al_2_O_3_ and water absorbed by KBr sampling pellet [15]. A weak absorption in 2800–3000 cm^−1^ (C(sp^3^)–H_n_) is due to organic contaminations absorbed from atmospheric ambient. It is worth noting that after annealing of pristine alumina at temperature up to 600 °C, the only change in FTIR spectrum was disappearance of C–H-related band at 2800–3000 cm^−1^.

Some additional absorption bands appeared after chemical treatment (Fig. 1, spectrum 2). Absorption band in spectral range 2800–3000 cm^−1^ (C(sp^3^)–H_n_) became much stronger and now is accompanied by absorption at 3000–3100 cm^−1^ (C(sp^2^)–H_n_) due to hydrogen bonded to benzene rings. Benzene rings of phenyl groups give rise to the narrow bands at 1136, 1430, and 1590 cm^−1^ (C=C vibration in benzene rings) as well as to the “benzene fingers” at 1700–2000 cm^−1^ due to overtone/combination vibrations in benzene rings. A strong and broad absorption band in range of 980–1200 cm^−1^ and centered at 1033 cm^−1^ is obviously due to siloxane bonds. A similar band was observed in phenyl-siloxane based polymers and associated with crosslinking of siloxane bonds into network [16, 17]. This band indicates formation of polymer-like siloxane precipitates on the surface of Al_2_O_3_ particles during chemical treatment procedure.

Most informative spectral ranges of the FTIR spectra of phenyl-alumina before and after annealing are shown in Fig. 2. IR bands related to benzene rings (1136, 1430, and 1590 cm^−1^) strongly reduced after annealing at 400 °С and at higher temperature (Fig. 2ab). Increase of the annealing temperature up to 600 °C results in high-frequency shift of Si–O related band from 1033 to 1070 cm^−1^ indicating transition from polymer-like to ceramic structure. Spectral position and shape of this band became typical for silicon oxide indicating formation of silica structural network presumably on the surface of aluminum oxide. It is approved by appearance of the shoulder at 450–460 cm^−1^ that can be assigned to Si–O–Si rocking vibrations.

Figure 2b shows that increasing of an annealing temperature causes decrease the intensity of the narrow absorption bands at 1430 and 1594 cm^−1^ and which is assigned to C=C stretching vibrations in phenyl rings. It is worth noting that traces of absorption by phenyl groups are detected up to highest annealing temperature. Destruction of benzene rings does not cause formation of amorphous pyrolytic carbon typically characterized by broad absorption band about 1600 cm^−1^. The absence of carbon precipitation can be explained by thermally activated carbon diffusion from surface inside Al_2_O_3_ particles during annealing in inert ambient. Carbon-doped aluminum oxide (Al_2_O_3_:C) is well known material widely used in dosimetry [11] and significant diffusion rate of carbon in Al_2_O_3_ is observed even at temperature as low as 400 °C [18].

Figure 2c illustrates evolution of C–H-related bands at 2800–3100 cm^−1^. It is seen that absorption bands at 2800–3000 cm^−1^, which corresponds to stretching vibrations of С(sp^3^)–H_n_ bonds in methyl groups as well as group of absorption bands at 3000–3100 cm^−1^, which corresponds to С(sp^2^)–H_n_ bonds in phenyl rings reduced strongly after annealing that is well consistent with thermally activated degradation of phenyl groups.

### Photoluminescence

**Fig. 3:**
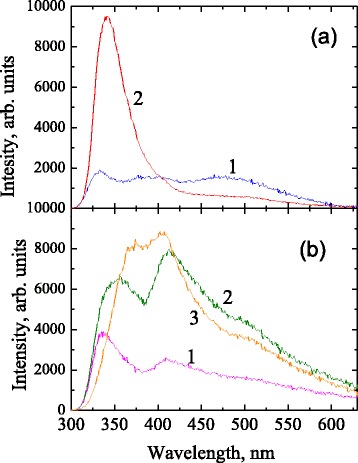
PL spectra under 290 nm excitation. **a** Pristine fumed alumina (*1*) and phenyl-alumina before annealing (*2*). **b** Phenyl-alumina after annealing at 400 °C (*1*), 500 °C (*2*), and 600 °C (*3*)

Pristine fumed alumina powder shows relatively weak broad band photoluminescence in spectral range 300–600 nm under 290 nm excitation (Fig. 3a, spectrum 1). Broad band is composed by at least three constituents with maxima at about 335, 390–400, and 470 nm. The band with a peak at 335 nm is likely due to oxygen vacancy with trapped electron (F^+^-centers) [9]. According to [19], the band with a maximum 390–400 nm can be associated with anion-cation vacancy pairs (P^−^-centers) or surface F^+^-centers (F_S_^+^-centers). The band with a maximum at 470 nm is possibly associated with F_2_-centers [20], but its correct identification needs further analysis.

Intense PL band with maximum at 340 nm appears in Al_2_O_3_ after chemical treatment (Fig. 3a, spectrum 2). This band is presumably associated with excimer states in closely located phenyl groups grafted to alumina surface [21–23]. Decomposition of phenyl groups during annealing leads to disappearance of this band (Fig. 3b). Emission band remains obviously multicomponent after annealing but increase of annealing temperature results in complicated evolution of intensity and spectral distribution. Increase of annealing temperature up to 500 °C leads to increasing of integrated PL intensity. It should also be noted that in the PL spectrum of sample annealed at a temperature of 500 °C it is observed low energy shift and broadening of UV peak (Fig. 3b, spectrum 2). Increasing of the annealing temperature up to 600 °C leads to a further shift of this band to 370 nm. Spectral position of emission peak at 410 nm and shoulder at 500 nm remained almost unchanged after annealing at 400–600 °C. As it was demonstrated by IR study, the structure of these samples can be represented as silica precipitates (presumably with carbon groups) on the surface of alumina nanoparticles. Such materials can be indicated as Al_2_O_3_/SiOC. Mechanism of formation of SiOC surface precipitates is believed to be similar to polymerization and structural crosslinking in polymer-derived SiOC ceramics obtained from phenyl-containing organosilicon precursor [17]. It is important also to note that pristine alumina annealed at the same conditions does not show any noticeable photoluminescence. Hence, it is reasonable to expect contribution of silica *and/or carbon*-related emission centers in visible PL band. Unfortunately, at present time we are not able to identify correctly the evolution of PL band of these samples.

**Fig. 4:**
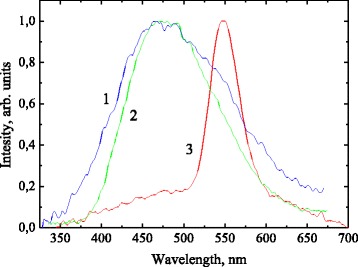
Normalized luminescence spectra under X-ray excitation: pristine alumina (spectrum 1), phenyl-alumina (spectrum 2), and phenyl-alumina after annealing at 400 °C (spectrum 3) at temperature 90 K

Using of UV emission allows exciting electron states with excitation energy far below the band gap of alumina and silica (commonly, it is electron states associated with bulk and surface defects). Alumina and silica have a very large band gap (9–10 eV), and examination of the effect of band-to-band excitation needs high-energy photons, for example X-ray excitation. Normalized PL spectra of the pristine alumina (spectrum 1), phenyl-alumina (spectrum 2), and phenyl-alumina (spectrum 3) annealed at 400 °C exited by X-ray at 90 K are illustrated in Fig. 4. PL spectra of pristine alumina and phenyl-alumina are quite similar representing broad band with maximum intensity about 470 nm. No detectable PL was observed at room temperature. Spectral similarity of the bands in pristine and chemically modified samples allows assigning this band to emission from alumina-related centers. Excimer PL in phenyl groups seems to be not excited by high-energy radiation. A narrow and almost symmetrical PL band centered at about 550 nm appears in the spectrum of the sample of the phenyl-alumina after annealed at 400 °C (spectrum 3). A weak but well pronounced broad PL background band is also observed. The origin of this broad background presumably associated with alumina structural network.

Taking into account that (1) narrow green PL band is observed only under X-ray excitation (i.e., high energy of excitation photon) and (2) formation of silica structural network after annealing at 400 °C it is reasonable to assign this emission band to self-trapped exciton in silica structure. Spectral position of PL band is well consistent with that reported in [24].

## Conclusions

Al_2_O_3_:SiOC nanocomposites were synthesized using thermal treatment of fumed alumina nanopowder modified by phenyltrimethoxysilane. Hydroxyl groups on the surface of alumina nanoparticles were replaced with phenylsiloxane groups followed by annealing in temperature range 400–600 °C. It is demonstrated that increase of annealing temperature results in pyrolysis of phenyl groups and formation of silica precipitates. No carbon precipitation was detected after pyrolysis of organosilicon groups. It is suggested that development of photoluminescence after thermal treatment is due to formation of carbonized silica on the surface of alumina particles.

## Abbreviations

Al_2_O_3_:C: Carbon-doped aluminum oxide
Al_2_O_3_:SiOC: Alumina/organosilicon nanocomposite
F^+^-center: Oxygen vacancy with trapped electron
F_2_-center: Two adjacent F-centers
F-center: Oxygen vacancy with two trapped electrons
F_S_^+^-center: Surface analog of F^+^-center
FTIR: Fourier transform infrared
IR: Infrared
P^−^-center: Anion-cation vacancy pairs
PhTMS: Phenyltrimethoxysilane
SiO_2_:C: Carbonized nanocomposite materials based on silica
